# Pivotal role of a conserved histidine in *Escherichia coli* ribonuclease HI as proposed by X-ray crystallography

**DOI:** 10.1107/S2059798322000870

**Published:** 2022-02-23

**Authors:** Zengwei Liao, Takuji Oyama, Yumi Kitagawa, Katsuo Katayanagi, Kosuke Morikawa, Masayuki Oda

**Affiliations:** a Graduate School of Life and Environmental Sciences, Kyoto Prefectural University, 1-5 Hangi-cho, Shimogamo, Sakyo-ku, Kyoto 606-8522, Japan; b Graduate School of Agricultural and Life Sciences, The University of Tokyo, 1-1-1 Yayoi, Bunkyo-ku, Tokyo 113-8657, Japan; c Faculty of Life and Environmental Sciences, University of Yamanashi, 4-4-37 Takeda, Kofu, Yamanashi 400-8510, Japan; d Faculty of Life and Environmental Sciences, Kyoto Prefectural University, 1-5 Hangi-cho, Shimogamo, Sakyo-ku, Kyoto 606-8522, Japan; e Graduate School of Intergrated Sciences for Life, Hiroshima University, 1-3-1 Kagamiyama, Higashi-Hiroshima, Hiroshima 739-8526, Japan; f Graduate School of Biostudies, Kyoto University, Yoshida-konoemachi, Sakyo-ku, Kyoto 606-8501, Japan

**Keywords:** X-ray crystallography, metallo­enzymes, endonucleases, *Escherichia coli*, ribonuclease HI, conserved histidine

## Abstract

The ribonuclease (RNase) H family of enzymes catalyze the specific cleavage of RNA strands of RNA/DNA hybrid duplexes and play an important role in DNA replication and repair. Since the first report of the crystal structure of RNase HI, its catalytic mechanisms, which require metal ions, have been discussed based on numerous structural and functional analyses, including X-ray crystallography. In contrast, the function of the conserved histidine residue (His124 in *Escherichia coli*) in the flexible loop around the active site remains poorly understood, although an important role was suggested by NMR analyses. Here, novel high-resolution X-ray crystal structures of *E. coli* RNase HI are described, with a particular focus on the interactions of divalent cations with His124 oriented towards the active site. The enzyme–Mg^2+^ complex contains two metal ions in the active site, one of which has previously been observed. The second ion lies alongside the first and binds to His124 in an octahedral coordination scheme. In the enzyme–Zn^2+^ complex a single metal ion was found to bind to the active site, showing a tetrahedral coordination geometry with the surrounding atoms, including His124. These results provide structural evidence that His124 plays a crucial role in the catalytic activity of RNase HI by interacting weakly and transiently with metal ions in the catalytic center.

## Abstract 2

X-ray crystal structures of *E. coli* ribonuclease HI in complex with Mg^2+^ ions or with a Zn^2+^ ion reveal the pivotal role of a highly conserved histidine residue.

## 1. Introduction

The ribonuclease H (RNase H) family of non-sequence-specific endonucleases hydrolyze the RNA strand of RNA/DNA hybrids (Majorek *et al.*, 2014 ▸; Hyjek *et al.*, 2019 ▸). Members of the RNase H family can be found in almost all organisms. They are mainly divided into two groups: RNase HI and RNase HII (or type 1 and type 2 in eukaryotes). Although their overall amino-acid sequences and tertiary structures are dissimilar, RNase HI and RNase HII share conserved active-center residues. RNase HI was concluded to have essential functions in nucleic acid metabolism, including removal of the RNA primers during replication (Holmes *et al.*, 2015 ▸; Al-Behadili *et al.*, 2018 ▸) and the regulation of R-loop homeostasis (Broccoli *et al.*, 2004 ▸; Cheng *et al.*, 2021 ▸). In the development of anti-HIV drugs in particular, remarkable efforts have been made to investigate the polymerase domain of HIV-1 reverse transcriptase (RT), despite the persisting problem of HIV-1 drug resistance. The RNase H domain of HIV-1 RT has also attracted substantial attention in drug-development research (Beilhartz & Götte, 2010 ▸; Esposito & Tramontano, 2014 ▸; Kankanala *et al.*, 2016 ▸).

RNase H cleaves the phosphodiester bonds of RNA in double-stranded DNA/RNA hybrids, leaving 3′-hydroxyl and 5′-phosphate groups at each cleaved site. This catalysis requires divalent cations such as Mg^2+^, Mn^2+^ and Zn^2+^. Interestingly, biochemical studies of RNase HI showed that its catalytic efficiency largely depends on the metal species and their concentrations (Goedken & Marqusee, 2001 ▸; Ando *et al.*, 2021 ▸). For example, RNase HI has the highest enzymatic activity in the presence of Mg^2+^ at an optimum concentration of ∼10 m*M*. Other metal ions such as Mn^2+^ and Zn^2+^ can also activate RNase HI, but with considerably limited efficiencies. In fact, the optimum concentration of Mn^2+^ is much lower than that of Mg^2+^. Notably, Ca^2+^ inhibits the enzymatic activity of RNase HI and thus is frequently used in crystallographic analyses of the complexes formed between RNase HI and DNA/RNA hybrid substrates.

Divalent cation-dependent phosphoryl-transfer reactions are highly ubiquitous among various endonucleases and exonucleases, most of which are notably involved in DNA repair or homologous recombination (Nishino & Morikawa, 2002 ▸). Therefore, these enzymes have attracted extensive interest from evolutionary aspects, with strong conservation in their main-chain folding and catalytic residue arrangements. In the case of RNase HI, the first report of the *Escherichia coli* RNase HI–Mg^2+^ complex described the observation of only one Mg^2+^ cation in the active site (Katayanagi *et al.*, 1993 ▸), whereas subsequent publications on the *E. coli* RNase HI–Mn^2+^ crystal structure (Goedken & Marqusee, 2001 ▸) and the *Bacillus halodurans* RNase HI–Mg^2+^–substrate crystal structure (Samara & Yang, 2018 ▸) reported the observation of two metal cations in the active site. Since then, the two-metal catalytic mechanism has been the mainstream hypothesis. However, molecular-dynamics studies of some DNA-repair enzymes and restriction endonucleases, which similarly require Mg^2+^, have suggested that one metal cation is sufficient for catalytic reactions (Dupureur, 2010 ▸).

In addition, crystallographic studies of RNase HI–substrate complexes are obliged to use mutant enzymes in which one of the essential catalytic residues is replaced by another residue. These mutations prevent the elucidation of the complete mechanism, including phosphoryl transfer and product release. In fact, the canonical two-metal-binding mechanism was subsequently extensively revised to a new model (Samara & Yang, 2018 ▸). This situation inspired us to reinvestigate more detailed crystal structures of metal-binding sites at higher resolutions in the absence of DNA/RNA hybrids.

**Figure 1:**
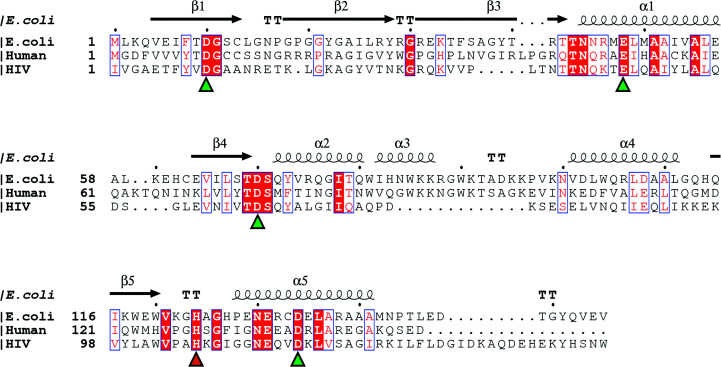
Sequence alignment of RNase HI from *E. coli* and human and the reverse transcriptase from human immunodeficiency virus (HIV). The secondary-structure depiction is based on the crystal structure of *E. coli* RNase HI (PDB entry 2rn2). Alignment was performed with *Clustal Omega* (Sievers *et al.*, 2011 ▸) and the figure was prepared with *ESPript* 3.0. Residues involved in the DEDD motif and the conserved histidine residue are indicated by green and orange triangles, respectively.

Besides the substrate and divalent cations, the negatively charged electrostatic field formed by the carboxyl side chains of Asp and Glu residues, usually referred to as the DEDD motif, is well known to be a key player in RNase HI function. Structural analyses found that the DEDD motif coordinates the divalent cation at the active site, acting as metal ligands (Katayanagi *et al.*, 1992 ▸), and constitutes the pivotal scaffold for the catalytic reaction (Kanaya *et al.*, 1990 ▸). In addition to the highly conserved DEDD motif, a histidine adjacent to the active site (His124 in *E. coli* RNase HI) in the loop structure between β5 and α5 (Fig. 1 ▸) has also frequently been discussed. This histidine has been reported to be significantly involved in proton transfer during the nucleophilic attack in the cleavage reaction (Oda *et al.*, 1993 ▸) and also to possibly be related to product release of RNase HI (Nowotny *et al.*, 2007 ▸). Consistently, an H124A mutation leads to a substantial loss of the nuclease activity (Kanaya *et al.*, 1990 ▸). Although ^1^H NMR analyses have suggested an indirect interaction between His124 and the DEDD motif (Oda *et al.*, 1993 ▸), the exact role of His124 during catalysis remains unclear due to the lack of an atomic structure of RNase HI showing a well defined conformation of His124. The previously reported crystal structures suggest that His124 could be present in multiple conformations in metal cofactor-unbound states (Yang, Hendrickson, Crouch *et al.*, 1990 ▸; Katayanagi *et al.*, 1990 ▸). Even in the crystal structure of RNase HI in complex with two Mn^2+^ ions in the active site, the His124-containing loop (residues 122–125) is disordered (Goedken & Marqusee, 2001 ▸). Therefore, more defined structural information is essential to clarify the functional role of His124.

Here, we report high-resolution crystal structures of *E. coli* RNase HI in complex with either Mg^2+^ or Zn^2+^. For each enzyme species, tetragonal and orthorhombic crystals suitable for high-resolution structure determination were obtained and various metal-binding states were observed. In the RNase HI–Mg^2+^ complex structure. we for the first time observed two Mg^2+^ ions in the catalytic site in a substrate-unbound state. Importantly, the coordination pattern differed between the RNase HI–Mg^2+^ and RNase HI–Zn^2+^ complexes. The observed clear electron density for His124 adjacent to coordinated metal cations allows us to more definitively discuss the role of His124 in the catalytic mechanism.

## 2. Materials and methods

### 2.1. Expression amd purification

*E. coli* RNase HI was expressed in the *rnhA*-deficient strain MIC3001 [F^−^, *supE44*, *supF58*, *lacY1* or (*lacIZY*)6, *trpR55*, *galK2*, *galT22*, *metB1*, *hsdR14* ( 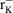 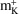 ), *rnhA339*::*cat*, *recB270*] with a heat-inducible expression system based on λ pR and pL promoters (Itaya & Crouch, 1991 ▸; Kanaya *et al.*, 1993 ▸). Cultivation was started at 30°C in LB medium. When the absorbance at 600 nm of the culture reached 0.8, the temperature of the incubator was increased to 42°C. After cultivation at 42°C for about 16 h, the cells were harvested and stored at −20°C until purification.

The cells were thawed and resuspended in 20 m*M* Tris–HCl pH 7.0, 10 m*M* NaCl, 1 m*M* EDTA, 1 m*M* dithiothreitol (DTT) and lysed by sonication. The cell debris was removed by centrifugation and the supernatant was loaded onto a SP Sepharose Fast Flow cation-exchange column (Cytiva) pre-equilibrated with the same buffer. The column was washed with 20 m*M* Tris–HCl pH 7.0, 100 m*M* NaCl, 1 m*M* EDTA, 1 m*M* DTT and the sample was eluted with a linear gradient of 100–300 m*M* NaCl. The eluted fractions containing RNase HI were pooled and loaded onto a HiLoad 26/600 Superdex 75 pg size-exclusion chromatography column pre-equilibrated with phosphate-buffered saline (PBS). The peak fractions containing RNase HI were dialyzed against 20 m*M* PIPES–NaOH pH 7.2 and were concentrated with Amicon Ultra-4 centrifugal filter units (Millipore). All purification procedures were performed at 4°C. The purity of all samples was checked with SDS–PAGE.

### 2.2. Crystallization

Concentrated RNase HI was diluted to a final concentration of 8 mg ml^–1^ in 20 m*M* PIPES–NaOH pH 7.2, 1 m*M* DTT with 50 m*M* MgSO_4_ or 1.5–2 m*M* ZnCl_2_. The original target of the research was to capture the transient bound state of RNase HI with adenosine monophosphate (AMP) or oligonucleotides; these substrates were also added to the sample in an RNase HI:substrate molar ratio of 1:5 or 1:10. Crystallization conditions were first screened with commercial screening kits (Crystal Screen and Crystal Screen 2 from Hampton Research) by the sitting-drop vapor-diffusion method at 20°C. 1 µl of the RNase HI sample was mixed with the same volume of reservoir solution and equilibrated against reservoir solution. The crystallization conditions were mainly optimized by adjusting the precipitant concentration. Optimization was carried out using either the hanging-drop or the sitting-drop method at 20°C by equilibrating a mixture of 2 µl RNase HI sample and 2 µl reservoir solution. Crystals appeared in 2–4 days and reached equilibrium in about one week. Most of the crystals had a thin needle-like shape with a typical thickness of around 25–50 µm. Although a significant amount of precipitation was observed when mixing RNase HI with the ZnCl_2_ solution, diffraction-quality crystals were obtained from several crystallization conditions. The optimized crystallization conditions are summarized in Table 1 ▸.

### 2.3. Diffraction data collection and processing

Crystals of suitable size were harvested, soaked in reservoir solution supplemented with 15% or 20%(*v*/*v*) glycerol and flash-cooled in liquid nitrogen. X-ray diffraction data were collected on BL45XU at the SPring-8 synchrotron-radiation facility (Yamashita *et al.*, 2018 ▸; Hirata *et al.*, 2019 ▸). Diffraction data were indexed, integrated and scaled using *XDS* (Kabsch, 2010 ▸). Data reduction was performed using *AIMLESS* from the *CCP*4 suite (Evans & Murshudov, 2013 ▸). Molecular replacement was performed using *Phaser* from the *Phenix* suite (Liebschner *et al.*, 2019 ▸). Refinement was repeated using *phenix.refine*, with manual modification in *Coot* (Emsley *et al.*, 2010 ▸). Data-collection and processing statistics are summarized in Table 2 ▸. Molecular graphics were obtained and analyses were performed with *UCSF Chimera* (Pettersen *et al.*, 2004 ▸).

### 2.4. Isothermal titration calorimetry measurements

Isothermal titration calorimetry (ITC) experiments (Supplementary Fig. S2) were performed on an iTC200 (Malvern Panalytical). ZnCl_2_ solution (1 m*M*) was titrated into RNase HI solution (0.1 m*M*) in 20 m*M* Tris–HCl pH 7.2 using a 40 µl syringe. Each titration consisted of a preliminary 0.4 µl injection followed by subsequent additions of 2.0 µl. Each corrected heat was divided by the number of moles of Zn^2+^ injected and was analyzed on the basis of the ‘one set of sites’ model using the *MicroCal Origin* 5.0 software supplied by the manufacturer. The binding stoichiometry (*n*), equilibrium dissociation constant (*K*_d_) and binding enthalpy change (Δ*H*) were directly calculated from the fitting procedure.

## 3. Results

### 3.1. Crystal structures of the *E. coli* RNase HI–Mg^2+^ complex

Although several types of substrates, such as trinucleotides and AMP, were added during the present crystallization trials, no clear electron density was identified corresponding to the substrates. These substrates were likely to be too small to produce stable complexes with RNase HI for crystallization. We obtained diffraction-quality crystals using similar reservoir conditions containing ammonium sulfate or polyethylene glycol (PEG). Based on the X-ray diffraction patterns, these crystals were found to belong to similar but different crystal forms with distinct unit-cell dimensions. In comparison with previously reported crystals, the molecular arrangements within the crystals showed a common feature in which the enzyme molecules form a similar dimer related by a twofold axis, as reported previously (Yang, Hendrickson, Kalman *et al.*, 1990 ▸; Katayanagi *et al.*, 1993 ▸).

**Figure 2:**
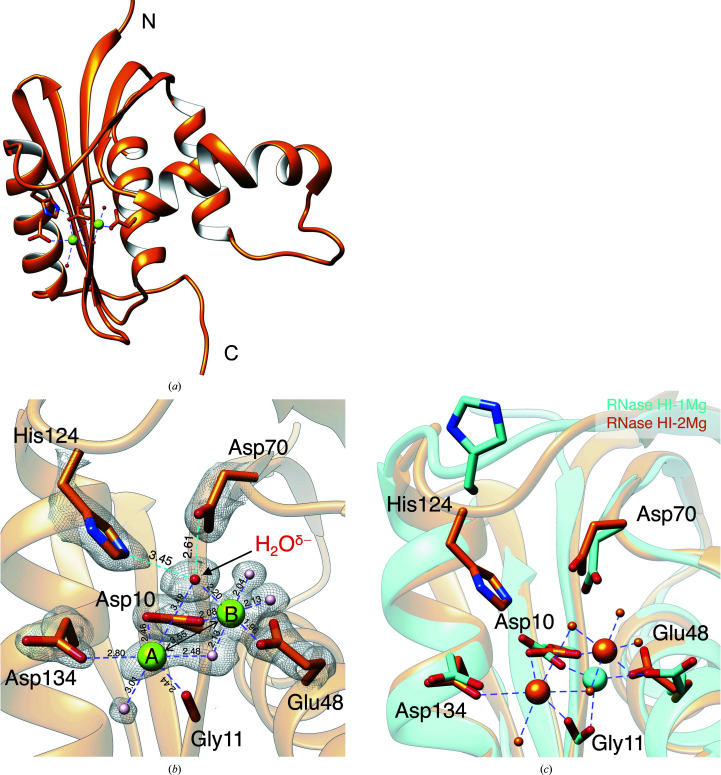
Mg^2+^ coordination around the active site of *E. coli* RNase HI. (*a*) Overall structure of *E. coli* RNase HI in complex with Mg^2+^. (*b*) The 2*F*_o_ − *F*_c_ electron-density map (1.3σ) of active-site residues and ligands involved in Mg^2+^–RNase HI coordination is shown as a gray mesh. Distances (in Å) between the Mg^2+^ ions and their coordinating ligands are indicated, as well as that for Mg^2+^ A–Mg^2+^ B. H_2_O^δ−^ is kept in the active site both by hydrogen bonds to Asp70 and the coordination by Mg^2+^ B (shown as light blue dotted lines). The Mg^2+^ ions are colored green, H_2_O^δ−^ red and the other surrounding waters light pink. (*c*) Superposition of the singly Mg^2+^-bound *E. coli* RNase HI (PDB entry 1rdd, cyan) and RNH_Mg_3 (orange). Mg^2+^ ions and water molecules are shown as large and small spheres in the same color as the protein. Putative coordination in RNH_Mg_3 is shown as purple dashed lines.

We obtained a total of five subunits from the three crystal forms. The root-mean-square distances among them for 155 corresponding C^α^ atoms are in the range 0.25–0.85 Å, indicating that they have essentially the same structure. Details of the nuances around the catalytic center are shown in Supplementary Fig. S1. In the RNH_Mg_2 structure an Mg^2+^ ion is bound in a different position to those in the other two structures, RNH_Mg_1 and RNH_Mg_3, which are very similar to each other. The exact reason for the observed variation in the binding of the Mg^2+^ ion is unknown. Hereafter, we describe chain *A* of RNH_Mg_3 in detail as a representative, because it is best ordered in the crystal. In the RNH_Mg_3 data set, two Mg^2+^ ions, designated metals A and B (Nowotny *et al.*, 2005 ▸; Nowotny & Yang, 2006 ▸), were observed in the vicinity of the active site, and both metals adopted a nearly ideal octahedral coordination with the surrounding acidic amino-acid residues and water molecules (Figs. 2 ▸ *a* and 2 ▸ *b*). Metal B was coordinated by four water molecules and the carbonyl groups of Asp10 and Glu48, while metal A was coordinated by three water molecules and the carbonyl groups of Asp10, Asp134 and Gly11. Two of the water molecules coordinating to the Mg^2+^ ions were shared. The distances between metal B and its coordination ligands were in the range 2.0–2.2 Å, whereas those of metal A ranged from 2.4 to 3.5 Å. A tightly bound metal B could be vital for precise binding to the phosphoryl group of a substrate. Conversely, loose interactions of metal A, which are reported to be pivotal to the nucleophilic attack of the coordinated water molecule, seem to correspond to mobility, which possibly benefits product release and the recruitment of a new Mg^2+^ ion. When comparing the behaviors of the Asp and Glu48 carboxyl side chains involved in Mg^2+^ coordination, that of the latter appears to be more functionally profound; the longer and more flexible side chain of Glu may allow metal B to form a more ideal octahedral coordination.

Fig. 2 ▸(*c*) shows a structural comparison of the observed doubly Mg^2+^-bound RNase HI (2Mg^2+^-RNase HI) with the previously reported singly Mg^2+^-bound enzyme. The Mg^2+^ ion present in the singly bound state is in a similar position to metal B in the doubly bound state in RNH_Mg_3, at a distance of 1.43 Å. The side chain of His124 observed in the present research is in a different orientation and N^ɛ^ of the imidazole group is located only 3.45 Å from the Mg^2+^-coordinated water molecule. Moreover, the unambiguous electron density of the His124 side chain observed in this research also proves the existence of the N^ɛ^–H_2_O interaction (Fig. 2 ▸ *b*).

### 3.2. Crystal structures of the *E. coli* RNase HI–Zn^2+^ complex

**Figure 3:**
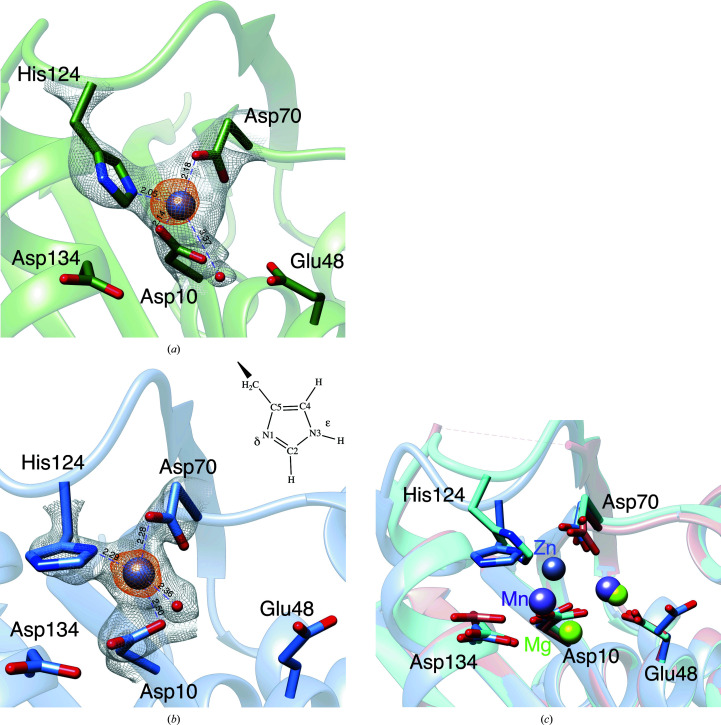
Zn^2+^ coordination around the active site of *E. coli* RNase HI: (*a*) RNH_Zn_1, (*b*) RNH_Zn_2. Zinc ions are shown as purple spheres. 2*F*_o_ − *F*_c_ maps (gray mesh, 1.3σ) of the RNase HI side chain and water molecule coordinated by Zn^2+^ and anomalous maps [orange mesh, 7σ in (*a*) and 6σ in (*b*)] of the Zn^2+^ ion are shown. Pseudo-bonds representing coordination are shown as purple dashed lines and the distances (in Å) between Zn^2+^ and coordinating ligand molecules are also given. (*c*) RNase HI–Mg (RNH_Mg_3, cyan), RNase HI–Mn (PDB entry 1g15, salmon) and RNase HI–Zn (RNH_Zn_1, cornflower blue) are superimposed. The loop structure of residue 122–125 in PDB entry 1g15 was disordered and not included in the structure model.

Structures of the Zn^2+^-bound enzyme were determined in tetragonal and orthorhombic crystal forms. In both forms, the RNase HI–Zn^2+^ complex structures indicated that a Zn^2+^ atom is bound to the active site (Figs. 3 ▸ *a* and 3 ▸ *b*). Consistent with this structural observation, ITC measurements indicated that the enzyme binds one Zn^2+^ ion per molecule, with an estimated *K*_d_ of 1.09 µ*M* (Supplementary Fig. S2).

The Zn^2+^ ion in both structures was in a tetrahedral co­ordination with the imidazole group of His124, the carboxyl groups of Asp10 and Asp70 and a water molecule. However, the orientations of the coordinated His124 and Asp10 were different from each other. In the RNH_Zn_1 structure (Fig. 3 ▸ *a*) the N^ɛ^ atom of the His124 imidazole group forms a coordination bond with the Zn^2+^ ion, whereas in the case of RNH_Zn_2 (Fig. 3 ▸ *b*) the N^δ^ atom participates in the tetrahedral coordination.

The p*K*_a_ of *E. coli* RNase HI His124 in the metal-free state was reported to be around 7, according to the results of ^1^H-NMR analysis (Huang & Cowan, 1994 ▸). The pH values of the crystallization conditions of RNH_Zn_1 and RNH_Zn_2 were 4.6 and 5.6, respectively, and thus these lower pH values do not appear to explain the difference. Presently, it seems more likely that differences in ionic strength may have caused the distinct orientations of the imidazole side chain on the flexible loop.

## 4. Discussion

### 4.1. The current 2Mg^2+^-RNase HI structure supports a previously proposed carboxylate–hydroxyl relay mechanism including His124

**Figure 4:**
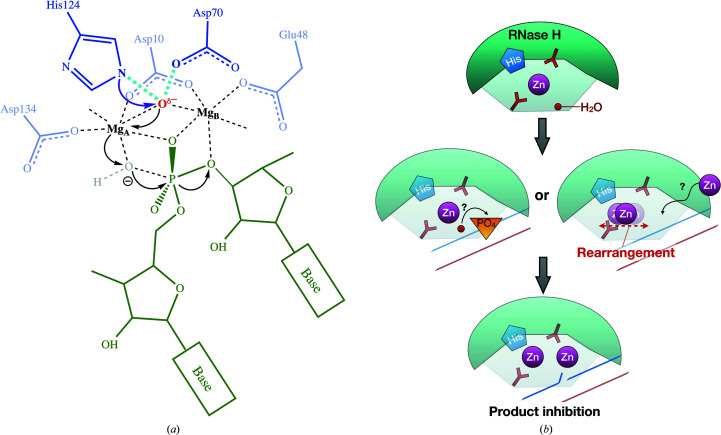
Possible carboxylate–hydroxyl relay mechanism including His124. (*a*) Schematic drawing of the proposed His124-dependent proton-relay pathway. The amino-acid residues, Mg^2+^ ions and H_2_O^δ−^ water molecule are drawn based on the current crystal structure. A substrate is drawn on the basis of the hypothetical structure in comparison with the human RNase H1–RNA/DNA hybrid complex (PDB entry 2qkk). A putative nucleophilic attack water (gray) is also indicated. (*b*) A putative mechanism for the formation of an intermediate ternary complex after the RNase HI–Zn^2+^ complex. The Zn^2+^-coordinated water molecule resembling that in RNH_Zn_1 and RNH_Zn_2 is indicated in the first step of the scheme.

When considering the Mg^2+^-coordinated H_2_O^δ−^ adjacent to Asp70, it is likely that this water molecule is anchored by both a coordination bond to Mg^2+^ and a hydrogen bond to the carboxyl group of Asp70 (Fig. 2 ▸ *b*). The side chain of His124 is also located close to Asp70 and H_2_O^δ−^. It has been reported that the resonance corresponding to C2H of His124 shifts significantly on the addition of Mg^2+^ in ^1^H-NMR analysis (Huang & Cowan, 1994 ▸). In the present research, the electron density for the imidazole side chain was very clear (Fig. 2 ▸ *b*; Supplementary Fig. S3), even though His124 is located in a flexible loop structure. Therefore, it is reasonable to infer that this side chain is firmly fixed by adjacent amino-acid residues or water molecules. H_2_O^δ−^ made relatively strong interactions with Mg^2+^ at the B position and Asp70, with O–*X* distances of 2.20 and 2.61 Å, respectively, while the O–N distance of 3.45 Å between the imidazole group of His124 and H_2_O^δ−^ suggests a weaker hydrogen bond. These detailed views are in good agreement with a scenario involving a His124→H_2_O→Mg^2+^→H_2_O→PO_4_ carboxylate–hydroxyl relay mechanism (Fig. 4 ▸ *a*), as proposed by the previous NMR analyses (Oda *et al.*, 1993 ▸).

### 4.2. The strong Zn^2+^ binding to the active site of RNase HI may explain the product-release inhibition of the previously reported enzyme–2Zn^2+^–RNA(nicked)/DNA ternary complex

The Zn^2+^-bound water molecule appears to be a critical component of a catalytic zinc site because it can either be ionized or polarized to aid the catalytic reaction or be displaced by the substrate (McCall *et al.*, 2000 ▸). Previous mass-spectroscopic and biochemical analyses (Ando *et al.*, 2021 ▸) indicated that cleavage of RNA^8^/DNA^8^ hybrid substrates occurs in the presence of Zn^2+^. The ternary-complex products included RNase HI–Zn^2+^–RNA^4 or 5^/DNA^8^ and RNase HI–2Zn^2+^–RNA^8^(nicked)/DNA^8^, indicating inhibition of product release. The present crystal structures in the absence of substrate show that the single Zn^2+^ ion is located at a considerably different position from the two Mg^2+^ sites (Fig. 3 ▸ *b*). Thus, it appears to be too difficult to propose even a tentative catalytic scheme, while the Zn^2+^ ion certainly plays a critical roles in the cleavage reaction. However, one water molecule coordinated to the Zn^2+^ ion is intriguing in terms of its possible involvement in substrate hydrolysis, for instance, as a candidate for a nucleophilic molecule (McCall *et al.*, 2000 ▸). Presumably, substrate binding to the enzyme would cause some structural rearrangements and the concomitant introduction of the second Zn^2+^ into the catalytic center. The two Zn^2+^ ions, with much stronger coordination bond formation, would prevent the release of cleaved products (Fig. 4 ▸ *b*). Further analyses would be required to propose a clearer catalytic mechanism for Zn^2+^-bound RNase HI, including knowledge of coordination chemistry based on ternary-complex structure analyses.

### 4.3. Structural variation of His124 accompanied by metal binding may imply metal movements coupled to conformational change of the enzyme during the cleavage reaction

Structural comparison of RNase HI in complex with Mg^2+^, Mn^2+^ or Zn^2+^ (Fig. 3 ▸ *c*) showed obvious differences in the Me^2+^ (Mg^2+^, Mn^2+^ or Zn^2+^) at the A position adjacent to Asp134 and His124, while the Me^2+^ at the B position near Glu48 did not substantially change in the case of Mn^2+^ or Mg^2+^. It is particularly notable that the imidazole group of His124 has the possibility of interacting either directly with Zn^2+^ or indirectly with Mn^2+^ and Mg^2+^. This differing binding property can be ascribed to coordination-bond formation and electrostatic interaction abilities. Although RNase HI is not a sequence-specific endonuclease, cleavage sites of the same substrate vary depending on the types of metal cations (Ando *et al.*, 2021 ▸). The deviation of Me^2+^ at the A position may induce the substrate to fit into RNase HI at various positions, thereby generating different cleavage sites. In fact, the RNase HI–Zn^2+^–substrate ternary complex formed product-inhibition intermediates (Ando *et al.*, 2021 ▸), possibly due to the strong binding of the Zn^2+^ ion to the enzyme, as shown by our ITC measurements (Supplementary Fig. S2). This result contrasts with the finding that wild-type RNase H never produces such intermediates at some 10 m*M* level concentrations of Mg^2+^. Presumably, the His124–H_2_O^δ−^ interaction could maintain the Mg^2+^–His124 interaction with moderate affinity without preventing product release. This notion highlights that the mobility of metal ions during the cleavage reaction may be a critical factor which could be determined by the surrounding protein atoms in the active site.

### 4.4. Pivotal role of His124 in product release

Although His124 in *E. coli* RNase HI is not conserved in *B. halodurans* RNase H (Nowotny *et al.*, 2005 ▸), Glu188 in *B. halodurans* RNase H also coordinates a metal ion and functionally resembles His124 in *E. coli* RNase HI. The authors suggested the significance of Glu188 in product release, and also reported the similar possibility that the imidazole ring of His264 in *H. sapiens* mitochondrial RNase H1 may clash with the cleaved 5′-phosphate to facilitate dissociation of the product (Nowotny *et al.*, 2007 ▸). However, based on the results of the present research, we suppose that the role of His124 is more likely to be as a switch than as a physical ejector; the random movement of the His124-containing loop seems to be too inefficient for a highly active enzyme such as *E. coli* RNase H. In fact, the reported crystal structures of RNase H–substrate complexes revealed extensive interactions between the substrate backbone and RNase H, including them in the canonical ‘phosphate-binding pocket’ (Nowotny *et al.*, 2007 ▸). More detailed analyses will be required to clarify the exact role of His124 in *E. coli* RNase and the corresponding residues in related enzymes.

## 5. Conclusion

We have described two novel high-resolution crystal structures of *E. coli* RNase HI, in which the structure of the conserved His124 in the flexible loop was well defined in the electron-density map by interaction with the divalent metal ions. These findings provide a new structural view that indicates a possible pivotal role of this histidine residue during the cleavage reaction.

## Supplementary Material

PDB reference: ribonuclease HI, complex with two Mg^2+^ ions, 7vsa

PDB reference: complex with one Zn^2+^ ion (His124 N^δ^ binding), 7vsb

PDB reference: complex with one Mg^2+^ ion, 7vsc

PDB reference: 7vsd

PDB reference: complex with one Zn^2+^ ion (His124 N^ɛ^ binding), 7vse

Supplementary Figures. DOI: 10.1107/S2059798322000870/ji5026sup1.pdf

## Figures and Tables

**Table 1:** Crystallization conditions

Sample ID	Crystallization condition	Ligand and/or substrate
RNH_Mg_1	0.2 *M* sodium acetate trihydrate, 0.1 *M* sodium cacodylate trihydrate pH 6.5, 30%(*w*/*v*) polyethylene glycol 8000	0.1 *M* magnesium sulfate, 10 m*M* AMP
RNH_Mg_2	0.2 *M* ammonium acetate, 0.1 *M* sodium citrate tribasic dihydrate pH 5.6, 30%(*w*/*v*) polyethylene glycol 4000	0.1 *M* magnesium sulfate, 10 m*M* AMP
RNH_Mg_3	0.2 *M* ammonium sulfate, 0.1 *M* MES monohydrate pH 6.5, 30%(*w*/*v*) polyethylene glycol monomethyl ether 5000	0.1 *M* magnesium acetate, 5 m*M* AMP
RNH_Zn_1	0.2 *M* ammonium sulfate, 0.1 *M* sodium acetate trihydrate pH 4.6, 30%(*w*/*v*) polyethylene glycol monomethyl ether 2000	2 m*M* zinc chloride
RNH_Zn_2	0.1 *M* sodium citrate tribasic dihydrate pH 5.6, 20%(*v*/*v*) 2-propanol, 20%(*w*/*v*) polyethylene glycol 4000	1.5 m*M* zinc chloride, 2 m*M* substrate (p-CCU)

**Table 2:** Data-collection and refinement statistics Values in parentheses are for the highest resolution shell.

	RNH_Mg_1	RNH_Mg_2	RNH_Mg_3	RNH_Zn_1	RNH_Zn_2
Wavelength (Å)	1.0000	1.0000	1.0000	1.0000	1.0000
Resolution range (Å)	49.04–1.83 (1.895–1.830)	42.68–1.70 (1.761–1.700)	40.14–1.76 (1.823–1.760)	39.55–2.08 (2.154–2.080)	48.5–1.84 (1.906–1.840)
Space group	*P*4_3_22	*P*4_3_2_1_2	*P*222_1_	*P*222_1_	*P*4_3_2_1_2
*a*, *b*, *c* (Å)	62.00, 62.00, 80.14	85.36, 85.36, 77.80	61.64, 64.29, 80.29	60.65, 64.59, 79.10	86.17, 86.17, 80.11
α, β, γ (°)	90, 90, 90	90, 90, 90	90, 90, 90	90, 90, 90	90, 90, 90
Total reflections	177070 (10993)	362302 (17653)	202565 (11468)	124414 (9945)	291687 (16950)
Unique reflections	14238 (844)	32240 (1677)	32256 (1799)	19276 (1463)	26808 (1623)
Multiplicity	12.4 (13.0)	11.2 (10.5)	6.3 (6.4)	6.5 (6.8)	10.9 (10.4)
Completeness (%)	99.2 (98.3)	99.9 (99.9)	99.8 (100.0)	99.9 (99.9)	99.9 (100.0)
Mean *I*/σ(*I*)	11.2 (2.7)	10.4 (2.2)	14.9 (2.8)	13.6 (4.4)	17.1 (6.0)
Wilson *B* factor (Å^2^)	28.78	20.80	26.51	36.00	21.06
*R*_merge_	0.133 (1.417)	0.129 (0.950)	0.058 (0.596)	0.073 (0.354)	0.094 (0.384)
*R*_meas_	0.144 (1.533)	0.140 (1.049)	0.070 (0.709)	0.086 (0.415)	0.102 (0.424)
*R*_p.i.m._	0.056 (0.580)	0.056 (0.442)	0.038 (0.381)	0.046 (0.215)	0.041 (0.179)
CC_1/2_	0.993 (0.940)	0.997 (0.934)	0.997 (0.913)	0.997 (0.960)	0.996 (0.991)
CC*	1 (0.984)	1 (0.983)	1 (0.977)	1 (0.990)	1 (0.998)
Reflections used in refinement	14181 (1363)	32099 (3132)	32198 (3157)	19253 (1886)	26709 (2613)
Reflections used for *R*_free_	705 (69)	1591 (168)	1560 (156)	898 (89)	1293 (140)
*R*_work_	0.2027 (0.3034)	0.1865 (0.2492)	0.2002 (0.3080)	0.2307 (0.3320)	0.1852 (0.2007)
*R*_free_	0.2495 (0.3135)	0.2305 (0.2833)	0.2326 (0.3873)	0.2773 (0.3291)	0.2215 (0.2330)
No. of non-H atoms					
Total	1362	2668	2675	2615	2666
Macromolecules	1267	2501	2522	2498	2516
Ligands	12	13	31	28	2
Solvent	83	154	122	89	148
Protein residues	155	310	310	310	310
R.m.s.d., bonds (Å)	0.005	0.016	0.006	0.003	0.006
R.m.s.d., angles (°)	0.63	1.52	0.81	0.61	0.76
Ramachandran favored (%)	98.04	95.75	99.67	98.04	99.67
Ramachandran allowed (%)	1.96	3.92	0.33	1.96	0.33
Ramachandran outliers (%)	0.00	0.33	0.00	0.00	0.00
Rotamer outliers (%)	2.29	5.45	2.69	8.53	2.30
Clashscore	2.76	11.05	2.96	4.59	3.39
Average *B* factor (Å^2^)					
Overall	37.26	38.18	35.70	43.26	29.73
Macromolecules	37.00	38.42	35.31	43.09	29.62
Ligands	46.54	29.26	54.53	61.08	67.91
Solvent	39.83	34.96	38.88	42.35	31.14
PDB code	7vsc	7vsd	7vsa	7vse	7vsb
