# Supplementary material for: Pivotal role of a conserved histidine in Escherichia coli ribonuclease HI as proposed by X-ray crystallography
Source: Acta Crystallogr D Struct Biol. 2022 Feb 23;78(Pt 3):390–8. doi: 10.1107/S2059798322000870 (PMC8900815; doi:10.1107/S2059798322000870)
Supplement: Supplementary file 1 [file d-78-00390-sup1.pdf]

STRUCTURAL
BIOLOGY

Volume 78 (2022)

Supporting information for article:

Pivotal role of a conserved histidine in *Escherichia coli* ribonuclease HI as proposed by X-ray crystallography

Zengwei Liao, Takuji Oyama, Yumi Kitagawa, Katsuo Katayanagi, Kosuke Morikawa and Masayuki Oda

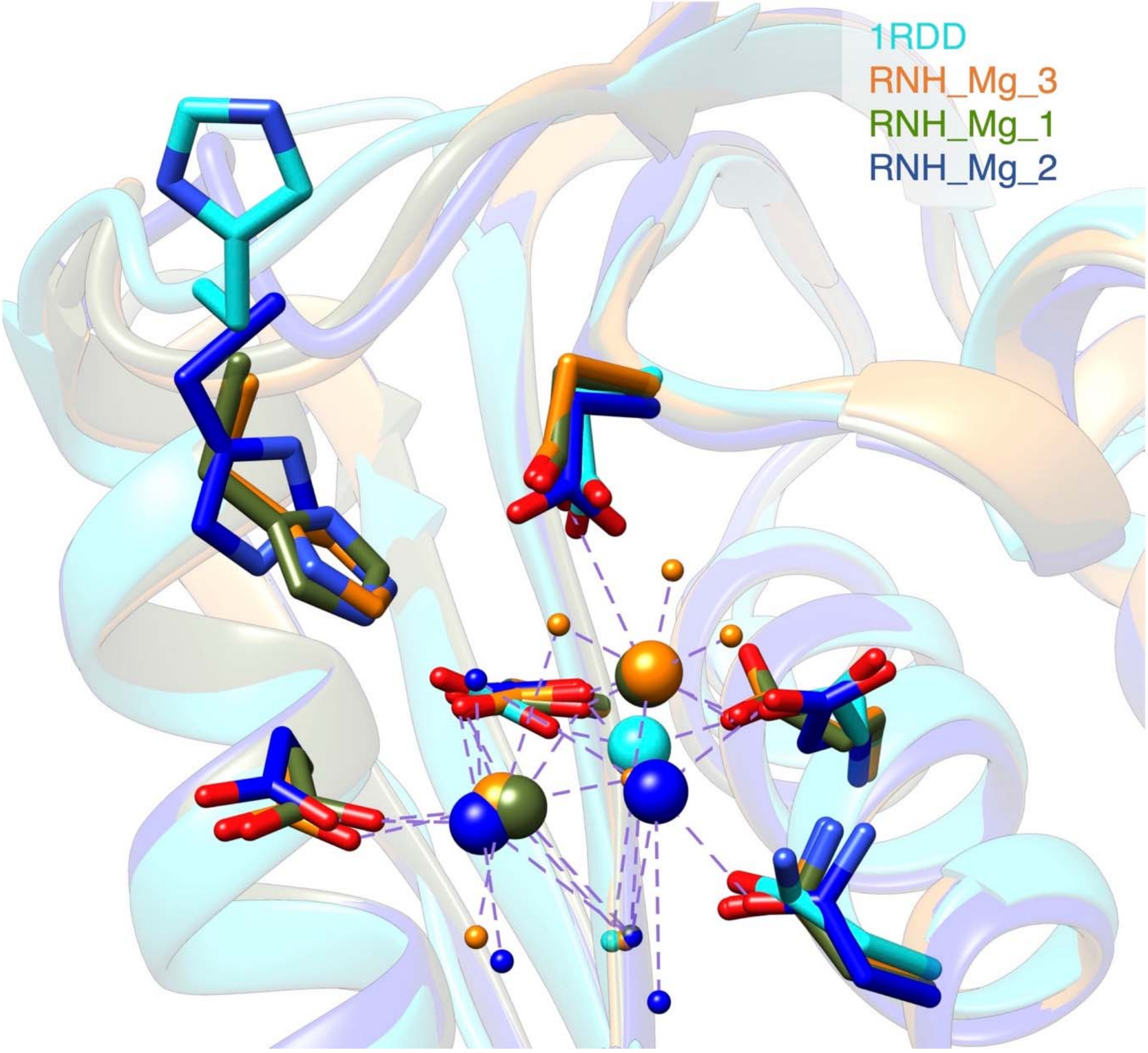

Figure S1 Comparison of Mg^{2+} coordination patterns in the *E. coli* RNase HI crystal structures. Dark olive green: RNH_Mg_1, blue: RNH_Mg_2 Orange: RNH_Mg_3, cyan: one Mg^{2+} bound *E. coli* RNase HI (PDB entry 1RDD). Note that one Mg^{2+} position (upper right) of two Mg^{2+} sites looks like a single metal, because of nearly perfect overlapping.

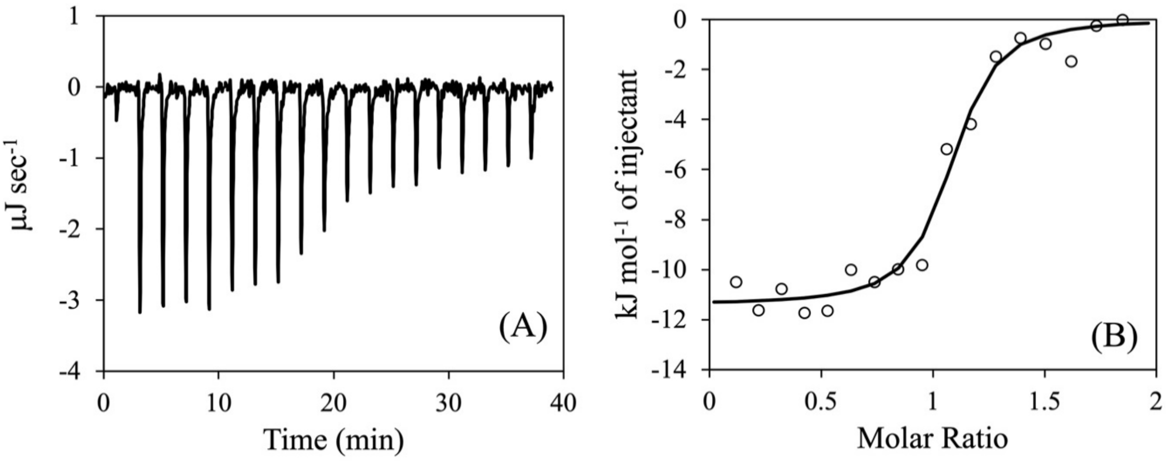

Figure S2 ITC measurements of the interaction between RNase HI and Zn^{2+}. (A) The $ZnCl_2$ solution was injected into the RNase HI solution. (B) Integrated data was fitted using one-site model. The three independent experiments gave n = 0.99 ± 0.15, K_d = 1.09 (± 0.60) μM, and ΔH = 11.7 (± 0.6) kJ mol^{-1}.

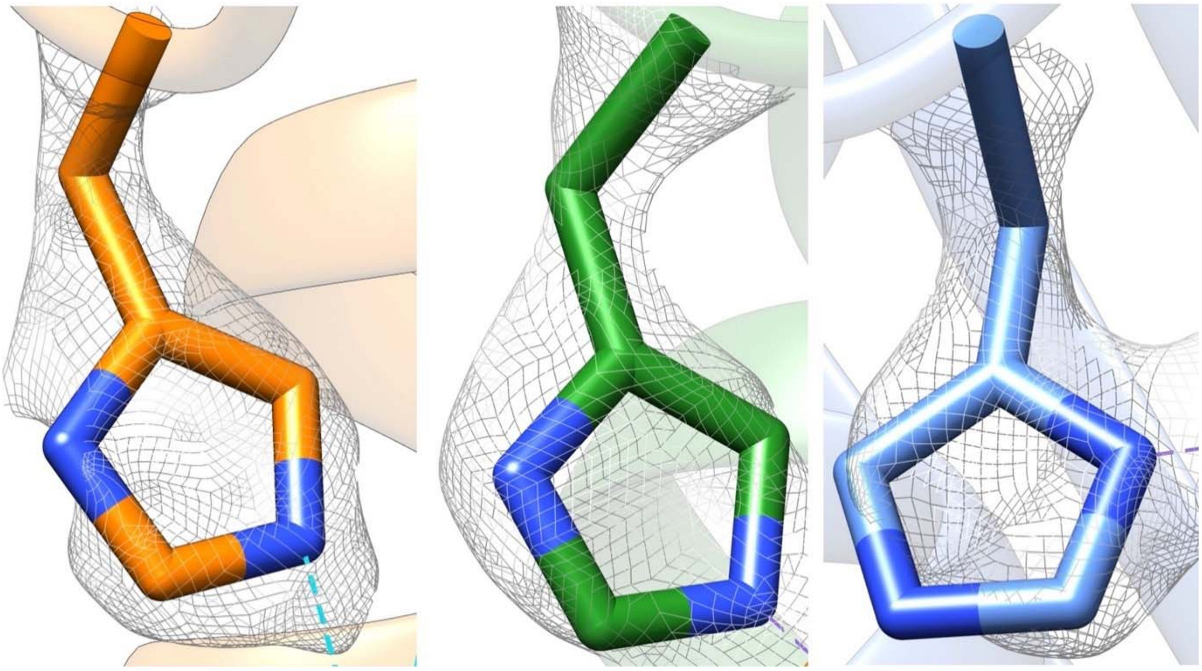

Figure S3 $2F_o$-F_c electron density maps (level: 1.3 σ) of His124 in RNH_Mg_3, RNH_Zn_1 and RNH_Zn_2 respectively. The His124 in each structure is zoomed in and the angle is adjusted to show the complete map of the whole imidazole sidechain.
